# Professional Merit

**Published:** 1852-10

**Authors:** S. D. Thompson

**Affiliations:** Providence, R. I.


### ARTICLE X.

*Professional Merit.* By S. D. THOMPSON, D. D. S., Providence, R. I.

PROFESSIONAL merit is a subject that is often touched upon, and a point we find many stickling for. Every man loves to be thought well of, let his profession, or calling, be what it may. Singular indeed must be that individual whose feelings do not sympathise with the voice of commendation, or who does not feel a peculiar satisfaction when his name is mentioned in a commendatory manner; this is as it should be, it is a satisfaction to the feelings, to know that our labor, or services, are appreciated, and that our toil has earned us something besides the dollar. A good name, a reputation for honor, for virtue, for integrity will stand when wealth has been scattered and its worshipers have crumbled into dust. The revered name of Washington will be written on the heart of hearts of every true American, so long as this republic shall stand, whilst the miser, with his hoards of pelf, shall cease to be remembered.

But to the point.

Since I have been conversant, in any manner, with the dental profession, and have learned the principles that have actuated the majority of the members of the American Society of Dental Surgery, I have admired the stand which they have taken. The standard set up by this associated body of dentists, (as far as my observations have extended,) is one of noble bearing, and waves proudly before the world. I have thought I could read written on its broad folds, sentiments like the following, "Death to empiricism," "Improvements free to the profession, and for the benefit of mankind," "No secrets or trickery in our practice," "We acknowledge no patent rights, medical or dental."

If we have not misinterpreted the principle of the American Society of Dental Surgery, they are broad, they are honorable, they are worthy to be followed and respected by every dentist throughout the land, and the world. These principles the world will acknowledge just and righteous.

We have said these are the principles of the majority, there has been, and are still among the members of that association, some who hug closely to secrets to patent rights, and who, by their advertisements, inform the world of the fact; they catch at every new discovery, invention, or patent, and blazon them in every possible manner, and then turn and ask if they are not deserving especial credit for their great efforts to benefit the public. When a physician, or dentist, purchases a patent right for some new agent, or article, for which he claims the right to use exclusive of all others in an entire state, we think he is taking a vast stride to professional distinction and fame.

We hear these same individuals prating about "empiricism,"

and condemning others for their avaricious motives, grasping at the "almighty dollar," having in view more the patent pocket, than his personal good. These we call *modest* professional men; in what class can such be ranked? they wish to be thought No. 1, and surely if their great bluster about improvements and great sacrifices are to be considered a part of professional merit, they should be A No. 1.

True merit, we have said in the outset, will usually meet its just deserts, and as we have imbibed that belief from occurrences in common every day life, we hope still to continue to see it verified.

In regard to the merit any individual deserves for peculiar improvements, (where they have been proved to be such,) we hope that there would not be an individual, claiming to be a member of the dental profession, who would wish to detract from such merit, or who would hesitate to make compensation for the benefit he might derive from any such invention or improvement. But let us have no professional patents which will enable one to secure to himself all such benefits, to the exclusion of all other members of the same profession throughout an entire state, thereby abridging its public usefulness.

